# Correction of phase offset errors in cardiovascular magnetic resonance using background subtraction from stationary tissue

**DOI:** 10.1186/1532-429X-13-S1-P213

**Published:** 2011-02-02

**Authors:** Nilanjana Misra, Amee M Shah, Wyman W Lai

**Affiliations:** 1 Columbia University Medical Center, NY, NY, USA

## Introduction

Phase offset errors from local eddy currents can lead to significant errors in flow assessment on clinical cardiovascular magnetic resonance (CMR) examinations. Phantom correction for phase contrast (PC) errors appears to improve accuracy in flow assessment, but requires additional time and is not free from technical limitations. Background subtraction using a region of stationary tissue has been proposed as an alternative method for the correction of phase offset errors.

## Purpose

To assess the validity of background correction using stationary tissue in the chest wall with stationary phantom correction for flow measurements in clinical practice.

## Methods

The clinical congenital CMR imaging database from July 2009 to May 2010 at our institution was searched for pts with: 1) no systemic-to-pulmonary shunt by echocardiography, 2) no valvular disease, and 3) normal ventricular volumes. Studies were performed on a GE Signa HDx 1.5T scanner, and PC flows measured with the resident FastCine PC pulse sequence (GE Healthcare, Milwaukee, WI). Breathe-though PC images were acquired perpendicular to the ascending aorta and main pulmonary artery. The flow sequences were repeated on a stationary fluid phantom to establish a baseline of zero velocity. The PC images were analyzed without and with phantom correction using GE ReportCard, and with background subtraction from stationary anterolateral chest wall tissue. The pulmonary-to-systemic blood flow ratio (Qp/Qs) was calculated for each method, as well as by volumetric analysis of SSFP short axis images. The methods were compared to each other using paired student t tests. Correlation coefficients were calculated for each method. No shunt was defined as Qp/Qs = 1.00 +/- 0.15.

## Results

There were 22 pts (median age 15.7 yrs, range 2 to 42 yrs) identified who met the study inclusion criteria. The average Qp/Qs (mean +/- SD) for No Correction was 1.27 +/- 0.34; Phantom Correction 1.03 +/- 0.15 (*p<0.05 versus No Correction); Stationary Tissue Correction 1.13 +/- 0.39; and Volumetric Analysis 0.97 +/- 0.09. Of the 22 pts with PC imaging, the correct diagnosis of no shunt was made on 12 pts without correction, 20 using phantom correction, and 11 using stationary tissue correction. By volumetric analysis, the diagnosis of no shunt was correctly made in 19 of 20 pts.

## Conclusions

Background subtraction using stationary tissue does not appear to be as accurate as phantom correction for PC flow assessment in clinical CMR studies. This finding should be assessed in laboratories using other CMR platforms.

